# Decision aid for prostate cancer screening in Brazil

**DOI:** 10.11606/s1518-8787.2022056003467

**Published:** 2022-03-29

**Authors:** Renata Oliveira Maciel dos Santos, Mirhelen Mendes de Abreu, Arn Migowski, Elyne Montenegro Engstrom

**Affiliations:** I Instituto Nacional de Câncer José Alencar Gomes da Silva Rio de Janeiro RJ Brasil Instituto Nacional de Câncer José Alencar Gomes da Silva. Rio de Janeiro, RJ, Brasil; II Universidade Federal do Rio de Janeiro Programa de Pós-Graduação em Clínica Médica Faculdade de Medicina Rio de Janeiro RJ Brasil Universidade Federal do Rio de Janeiro. Programa de Pós-Graduação em Clínica Médica da Faculdade de Medicina. Rio de Janeiro, RJ, Brasil; III Instituto Nacional de Câncer José Alencar Gomes da Silva Instituto Nacional de Cardiologia Rio de Janeiro RJ Brasil Instituto Nacional de Câncer José Alencar Gomes da Silva. Instituto Nacional de Cardiologia. Rio de Janeiro, RJ, Brasil; IV Fundação Oswaldo Cruz Escola Nacional de Saúde Pública Sergio Arouca Rio de Janeiro RJ Brasil Fundação Oswaldo Cruz. Escola Nacional de Saúde Pública Sergio Arouca. Rio de Janeiro, RJ, Brasil

**Keywords:** Prostatic Neoplasms, prevention & control, Mass Screening. Clinical Decision-Making, Use of Scientific Information for Health Decision Making, Physician-Patient Relations

## Abstract

**OBJECTIVE:**

To present the development and validation processes of a decision aid for prostate cancer screening in Brazil.

**METHODS:**

Study with qualitative-participatory design for the elaboration of a decision aid for prostate cancer screening, with the participation of a group of men and physicians inserted in primary health care in 11 Brazilian states. Evidence synthesis, field testing, and use in clinical scenarios were performed to adapt the content, format, language, and applicability towards the needs of the target audience in the years 2018 and 2019. The versions were subsequently evaluated by the participants and modified based on the data obtained.

**RESULTS:**

We elaborated an unprecedented tool in Brazil, with information about the tests used in the screening, comparison of their possible benefits and harms and a numerical infographic with the consequences of this practice. We verified the decision aid usability to assist in the communication between the doctor and the man in the context of primary health care, besides identifying the need for greater discussion about sharing decisions in clinical scenarios.

**CONCLUSION:**

The tool was easy to use, objective, and has little interference in consultation time. It is a technical-scientific material, produced by research, with the participation of its main target audience and which is available free of charge for use in Brazilian clinical scenarios.

## INTRODUCTION

Prostate cancer screening is widespread in clinical practice, although the best available evidence points to an imbalance between the harms and possible benefits of this practice^1^. The routine performance of the prostate specific antigen (PSA) and/or digital rectal examination in asymptomatic men results in many diagnoses and is followed by important damage to the quality of life of men^2^.

These harms result from false-positive results, which may need a biopsy to rule out cancer, which may cause pain, bleeding, and infection^2^. Other harms are related to overdiagnosis and overtreatment, which are, respectively, to diagnose and treat a cancer that would not threaten life and have as more common complications erectile sexual dysfunction and urinary incontinence^2,3^, in addition to emotional effects to men and family members.

Considering this information, since 2008, the National Cancer Institute (INCA) recommends that screening should only be done in men who spontaneously demand these tests and after shared decision-making, considering the risks and the uncertainty benefits^4,5^.

Shared decision-making is characterized by a collaborative process, where care options and their possible consequences are discussed to achieve the decision most appropriate for the person’s life context. This approach is especially relevant in situations with some degree of uncertainty in the relationship between harms and benefits. To facilitate share decision-making, international experiences recommend the use of clinical tools, called decision aid, to facilitate communication and deliberation. These tools are indicated, especially when the health outcomes encompass reasonable options, which people value differently^6^.

Decision aids may have various formats, including printed material, videos, or electronic devices, which stimulate the participation of users in share decision-making, facilitating the increase of knowledge on the discussed theme^7,8^.

To assist in the clinical communication of the implications of prostate cancer screening, a sensitive practice to individual preferences, we proposed to build a material that can be used during consultation in primary health care, the responsible level for cancer early detection in the Unified Health System^9^. The tool is a technical material of a Brazilian federal institution, available and distributed to primary health units in Brazil. Thus, this study aims to present the development and validation processes of a decision aid for prostate cancer screening in Brazil.

## METHODS

Following the International Patient Decision Aid Standards and previous experiences^10^, a three-step approach was used to develop and validate the decision aid, considering the target audience evaluation: healthy men and primary health care physicians. The study design was qualitative-participative, which advocates the integration of the subjects along the production of the study, contributing not only with consent or information, but modifying its direction from their knowledge and experiences^13,14^, as described in the following steps:

### Step 1: Tool Design

The first stage aimed to define the content and build the first version of the decision aid alongside experts. A literature review was conducted, with synthesis of the main available evidence on prostate cancer screening, considering clinical trials and systematic reviews on the effectiveness and consequences of screening. The search keywords was “prostate cancer”, “screening”, and “decision aid” in a combined way, in MEDLINE database, in Portuguese and English, for studies published in the last five years, without language filter.

The search recovered initially 1,200 references, resulted in 150 articles after title selection to check the scope and deleting 247 duplicates. Evaluating these 150 abstracts led to 20 articles for complete reading and analysis of evidence. Websites of research institutions that develop decision aids were also consulted as additional sources and examples of infographics^15,16^. The search subsidized the decision aid first version , constructed based on the contribution of six experts in cancer early detection and family medicine.

### Step 2: Calibration: Pilot Test.

The pilot test sought to adapt the format and language to the target audience: healthy men of mature age (approximately 40 years). The evaluation occurred by using a focus group, with 19 participants, employees of a public energy company, located in the municipality of Rio de Janeiro.

This scenario was chosen due to the company’s previous request to the federal institution for educational lectures on cancer for its workers. At the time, it was verified that the company had a considerable number of employees who fulfilled the inclusion criterion of the study. Thus, workers were invited to participate voluntarily in a research meeting during their work shift.

The meeting took place with audio recording, conducted by two facilitators, who worked on the research questions after individual and collective reading of the printed tool. The contributions were analyzed and incorporated into the second version, which followed the next stage of the study.

### Step 3: Feasibility and Use of the Tool

At this stage, the decision aid was applied by physicians in primary health care settings to a sample of their target audience, aiming to evaluate its applicability. Physicians were asked to use the tool in their consultation when some man demanded tests for prostate cancer screening. As inclusion criteria, physicians should have been working for at least one year in a primary health unit.

The physicians were invited by e-mail, telephone, or messaging application, after dissemination of the study by using the researchers’ network. The invitation explained the research objectives , the criteria for participation and asked interested parties to contact the lead researcher by e-mail or telephone. The interviewees were also asked to appoint other physicians to be invited.

The researchers sent 93 invitations, resulting in 42 responses from physicians interested in receiving and testing the tool. Weekly contacts were made with the participants to follow the study and request videoconference interview scheduling, after at least three uses. No doctor reported giving up participating in the study, however, 21 did not return follow-up messages and 6 claimed to have had no opportunity to use the tool. In the end, 15 physicians from 11 Brazilian states (Espírito Santo, Minas Gerais (2), Rio de Janeiro (2), Paraná, Paraíba (2), São Paulo, Santa Catarina, Rio Grande do Sul, Goiás, Rondônia, and Sergipe (2)) were interviewed.

Physicians evaluated the tool for the following criteria^11,12^:

- Content quality: examine the quality of information and the accuracy of scientific evidence;
- Layout: evaluate the layout and the infographic;
- Applicability: verify its application in the clinical context, use, acceptability, and interference in the consultation time.

The interviews were recorded, fully transcribed, and analyzed based on the previously described criteria. The suggestions for changes were incorporated into the decision aid, which went to the final version with text and layout adjustments and was later published as a technical product of a national institution specialized in cancer. The final version was sent to ten interviewed physicians who expressed a desire to receive the tool and four sent back comments.

This study followed the requirements of Resolutions 466/2012 and 580/2018 of the National Health Council regarding ethics in research with human beings, and was approved by CEP/ENSP, CAE: 12165019.4.0000.5240. All participants provided their agreement by signing the informed consent form.

## RESULTS

We developed the decision aid during the years 2018 and 2019 resulting in a two-page material, composed of explanatory texts on prostate cancer screening, the tests used, a comparative table on its harms and benefits, ending with a numerical infographic on the consequences of this practice. The technical content was based on the evidence of screening effectiveness ^2,15^, and we used the most current systematic review found, 2018^2^, as the main source of the infographic.

Following international models, the infographic starts with the estimated of screening in 1,000 men aged 55 to 69 years, followed-up throughout 13 years. The data presents the positivity rate of 10.2% for prostate cancer, followed by false-positive, which varied between 10.9% and 19.8% in studies, thus we used the 17.8% rate reported in five research centers monitored by the European randomized study of screening for prostate cancer (ERSPC)^18^. We also present the specific mortality reduction rates of 1.3% and the overdiagnosis rates of 50% based on this study^2,16^.

Regarding the consequences of treatment, we decided to highlight the potential harms of radical prostatectomy, since it is the most common type of treatment and the gold standard in the Brazilian guidelines for the diagnosis and treatment of prostate adenocarcinoma^19^. Thus, we presented the rates of 60% for sexual dysfunction, 20% for urinary incontinence and 0.5% for more severe complications and death^2,16^.

This first version of the tool went through the pilot test, being evaluated in a focus group composed of 19 men, aged between 38 and 72 years (mean = 51 years) and varied education levels and work function (Table 1).

**Table 1:** Profile of the men participating in the focus group of the pilot test, Rio de Janeiro, 2019.

	n	%
Group of men	19	
Age		
25 to 29 years old	0	0
30 to 39 years old	4	21
40 to 49 years old	3	16
50 to 59 years old	7	37
≥ 60 years old	5	26
Marital status		
Married	12	63
Single	6	32
Widower	1	5
Education level		
Primary education	4	21
High school	6	32
Unfinished higher education	1	5
Complete higher education	8	42

The group of men evaluation led changes in the amount of information (reduction of text and numerical data) and the infographic has been changed to a simpler and more linear presentation. We also modified some terms to facilitate understanding, but the content was considered appropriate and enlightening by most men. We also excluded reflective questions at the end of the material since mostly men considered them useless.

*This information is very important, here we can clearly see what can happen after taking these tests.* M15

*The material is very good, the flowchart [infographic] shows what can happen… having a complication, doing the surgery, then we think about the possibilities.* M2

The second version of the decision aid, a product of the modifications suggested by the group of men, was evaluated by physicians who used it between three and twelve times (mean = 6), aged between 25 and 46 years (mean = 35 years), more than half were female and most with a short time since graduation (< 5 years) (Table 2). Due to the geographical heterogeneity of the physicians’ practices, who worked in urban (60%) and rural (40%) areas, the decision aid was used in men of different profiles, most of them with low level of education and low socioeconomic status, aged around 50 years.

**Table 2:** Profile of the physicians who applied the tool, 2019.

	n	%
Physicians	15	
Age		
25 to 29 years old	8	53
30 to 39 years old	4	27
40 to 49 years old	2	13
50 to 59 years old	0	0
≥ 60 years old	1	7
Genre		
Female	9	60
Male	6	40
Time since graduation		
1 and < 2 years	6	40
≥ 2 and < 5 years	4	27
≥ 5 and < 8 years	4	27
≥ 8 years	1	7
Specialization		
Residency in family and community medicine	8	53
Other	1	7
Do not have	6	40
Region		
North	1	7
Northeast	4	27
Southeast	6	40
Midwest	1	7
South	3	19
Field of activity		
Urban	9	60
Rural	6	40

Most physicians (86%) considered the decision aid useful for clinical practice and easy to use, highlighting its objectivity and design as the main potentialities. They qualified the technical content as adequate and correct, without disagreement with the information presented.

The form of use was at the discretion of each physician, on the computer screen or printed, however, neither was superiority to the other, even when interspersed by the professional. However, some reports suggest greater practicality when the tool is available on the office computer.

*It’s easier when the material is already available on the computer we are using to attend and fill out the information in the medical records… when the patient talks about the subject you just open it and show them.* M5

On the other hand, two interviewees considered the decision aid as not very useful; the first states that the harms of screening should not be shown to men and suggested excluding this information; the second stated that the physician would not need to discuss the implications of the screening with the man since this practice is not recommended and suggested that the numerical data could hinder the understanding of men with low education level.[Fig f01] [Fig f02]

**Figure 1 f01:**
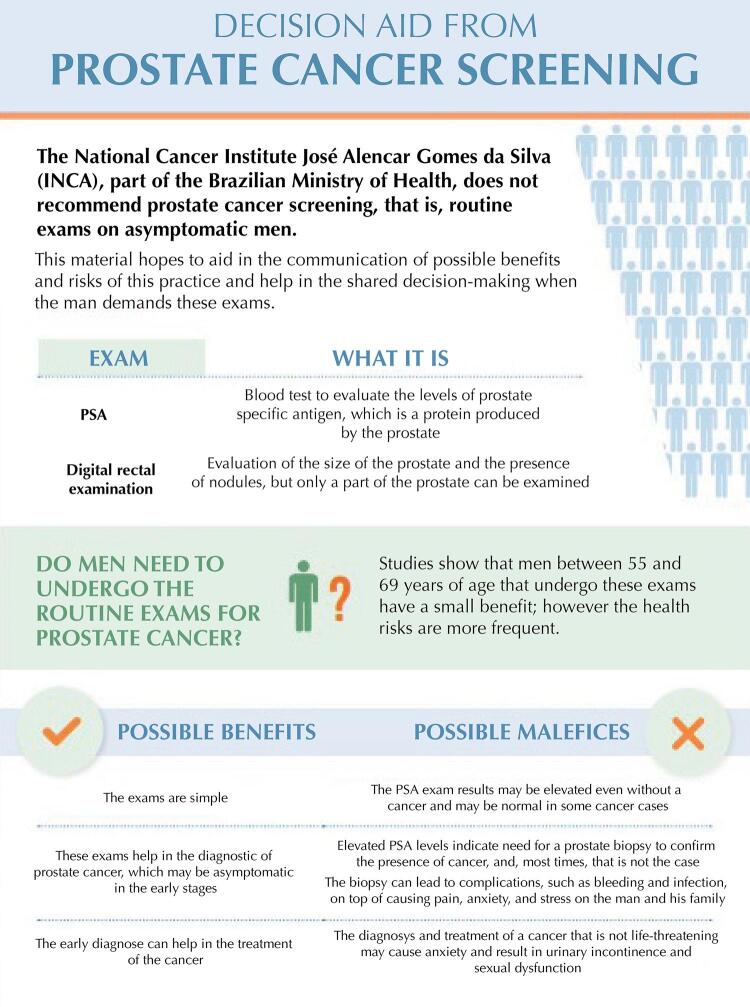
Final version of the decision aid tool for prostate cancer screening, page 1.

Source: Brazilian National Cancer Institute José Alencar Gomes da Silva. Available from: https://www.inca.gov.br/publicacoes/infograficos/ferramenta-de-apoio-decisao-no-rastreamento-do-cancer-de-prostata

**Figura 2 f02:**
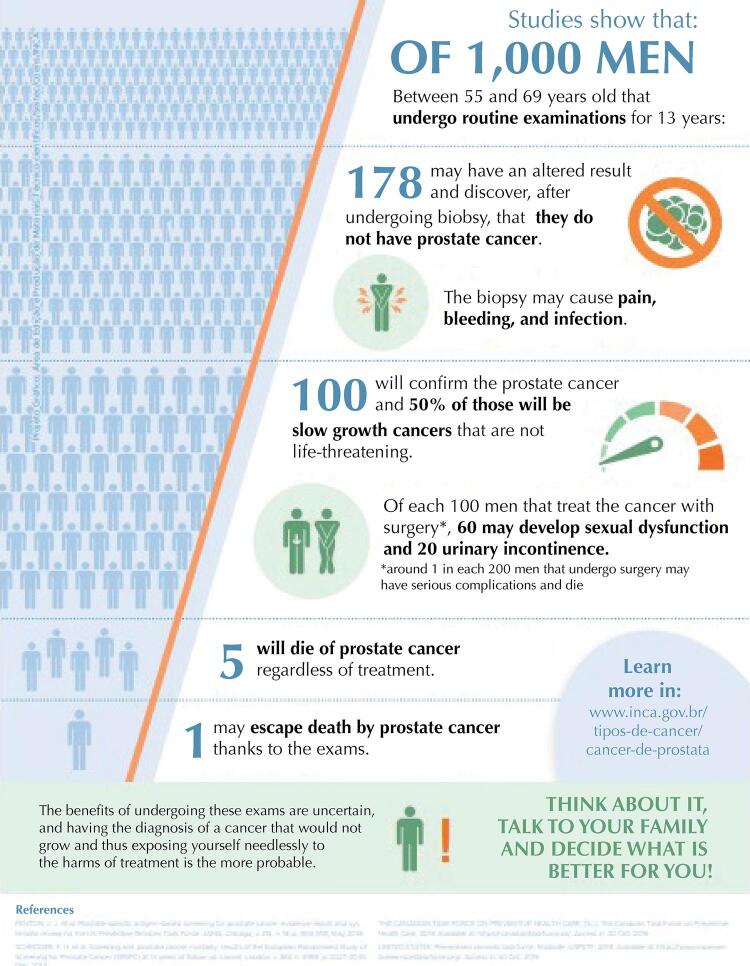
Final version of the decision aid tool for prostate cancer screening, page 2.

Source: Brazilian National Cancer Institute José Alencar Gomes da Silva. Available from: https://www.inca.gov.br/publicacoes/infograficos/ferramenta-de-apoio-decisao-no-rastreamento-do-cancer-de-prostata

*I thought it was good for the doctor, but I found it hard to explain … I felt that the information is not well understood and ended up taking a little longer to ask the person to look… translate it to him… for me it was good, helped me to systematize the information, but for the public itself, I don’t think it was.* M9

Regarding the interference in the consultation time, 53% of the physicians reported no change and 33% indicated a decrease in time, stating that the tool helps to remember all the points that should be addressed during the conversation, as highlighted in the speech below:

*It’s a lot that we have to say to the patient, I have to talk about diabetes, tuberculosis… when everything is already there, it helps a lot… and since I have little practice time, it makes it easier to remember everything I have to say.* M3

Most physicians (67%) reported that men chose to screen less after exposure to the decision aid, compared with previous visits. This change was attributed to a greater understanding of the harms, facilitated by the graphic resources and the institutional credibility conferred on the tool.

*It makes a difference when you have an institution behind it. I’m not the one saying, it’s not just the city doctor… There’s a credibility there, I feel safer to say it and the patient sees it written… it makes a lot of difference.* M8

Using the decision aid, physicians said they were more secure to transmit the information about the harms, due to the institutional support conferred by the material. Similarly, reports show that men were more confidence in not submit to the tests when they came across the tool. Physicians also reported that, without the decision aid, men knowing favorable information to screening often showed suspicion when presented to risks, especially those who had already undergone some previous examination.

*Some men get a little suspicious, especially the older ones, who have been doing these exams for years… I thought the material helped me show it to them, that there’s a science in it.* M6

On the other hand, the analysis of the interviews also showed that even with the tool the physician’s opinion about screening maintains a great weight in the decision since his beliefs can determine the choice of the man, as highlighted below:

*Here’s what happens if you do [the screening] … the risks… but I already show that it has benefits, that I think it’s better to do it, then most choose to do it, right…* M9

In this sense, as a negative point of the decision aid the physicians mentioned the frustration they feel when the man opts for a different decision from their own opinion and the time to incorporate a new tool in their routine. However, 13 interviewees (87%) answered that they would incorporate the tool into professional practice, and two of the physicians conditioned their use to changes in the layout and decrease in the amount of text, which were later incorporated into the final version.

The changes suggested by the physicians were excluding an explanatory text about the prostate cancer development, since 93% of the physicians considered it unnecessary, in addition to lengthening the material, and reduce the infographic text, heightening emphasis on numbers and graphic elements.

Another issue identified by the interviews was the unfamiliarity of some physicians on the decision aid, so we found necessary to add in the final version an instructional text about its purpose and the importance of discussing the risks and benefits with the man, when he demands the prostate cancer screening.

Physicians considered the final published version as easy to use and having appropriate language, content, attractive layout, and easy visualization of information. As external validation, we evaluated the tool using the International Patient Decision Aid Standards (IPDAS) to be incorporated into the International Inventory of Decision Aids^20^. The tool met all seven criteria that characterize a decision aid, the four criteria for a screening tool, six of the nine criteria for low risk of biases and 11 of the 13 quality criteria.

## DISCUSSION

The process of elaboration and validation of the decision aid showed its usefulness in the context of primary health care in Brazil, from the perception of its target audience. The tool was considered easy to use and adequate for communication between the physician and the man, regarding the deliberation on the possible risks and benefits of prostate cancer screening. Among the advantages associated with its use are the systematization of information, objectivity, and little interference in the consultation time.

The little interference and even the decrease in consultation time, perceived by some physicians in this study, is an important characteristic for implementation in clinical practice since health professionals constantly face the challenge of balancing many tasks in their time^21^. Systematic reviews that evaluated decision aids state that, depending on the context, type of intervention and way of use, consultation time may increase or decrease^7,8^. However, considering that the time spent in quality communication results in satisfaction and better health outcomes, among these, reducing return consultations due to the same causes^22,23^.

Regarding the decision aid acceptability, only two physicians interviewed indicated that they would not incorporate it into their clinical practice. One is contrary to screening and, thus, prefers not to discuss this possibility with the man and the other, being favorable, believes that the man should not be exposed to information about the harms. In both cases, the positions denote disagreement with the shared decision to screen for prostate cancer and not with the tool itself.

Shared decision-making is characterized by an interpersonal process, in which those involved collaborate to achieve the most appropriate action^22,23^. Although decision aid facilitate this approach^24^, by itself, they do not correspond to the whole process, since we could perceive some barriers to its development, such as the omission of the screening risks or the physician’s resistance to accept the man’s opinion , when different from his own.

Despite a protectionist intention, regarding the transmission of complex information, this attitude is contradictory with health ethics, which focuses on the individual right to know the consequences of interventions in their body, especially those with uncertain or unfavorable balance between harms and benefits^25^.

Moreover, the fact that physicians were unfamiliar with the decision aid reveals the need to stimulate the discussion about people participation in health decisions. The few Brazilian studies on shared decision-making point to the lack of implementation and institutionalization of this approach in clinical scenarios, which demonstrates the need to encourage this practice, with educational actions aimed at professionals^26^.

Similar results are found in studies that evaluated international decision aids, such as the need to invest in visual resources, less text to transmit the content^23,24^, difficulty of professionals in sharing information considered difficult to understand, and resistance to incorporate something new to clinical practice^24^.

The main limitations of our study are the impossibility of capturing the direct interference of the tool in the men’s decision, due to the difficulty in synchronizing the demand for care with data collection and the absence of comparison between care with and without the decision aid. Moreover, we recommend caution in generalizing the results since this is a qualitative study.

As a contribution, we consider the methodological process of development, which involved the target audience in the production of a technical-institutional material to be implemented in primary health care in Brazil, which can be replicated for the elaboration of other materials, and the decision aid itself, which can also be adapted to other scenarios and countries. The decision aid final version is available for free download at: https://www.inca.gov.br/publicacoes/infograficos/ferramenta-de-apoio-decisao-no-rastreamento-do-cancer-de-prostata, also distributed printed in small quantity.

We conclude that the decision aid developed in this study is useful for clinical practice, assisting in communication between men and physicians, besides favoring the discussion about the risks and benefits of prostate cancer screening. We highlight objectivity, graphic elements, and credibility as positive points, in addition to the little interference in the consultation time.

Finally, note that the participation of patients and physicians in the decision aid development made it possible to approximate the content and language to the needs of the target audience reaching, thus, greater correspondence with the material. However, evaluating the direct interference of the tool in the decision of men in future studies and making an implementation plan for the country, considering its regional heterogeneity regarding the organization of primary health care services, are necessary.
