# Supplementary material for: Decision aid for prostate cancer screening in Brazil
Source: Rev Saude Publica. 2022 Mar 29;56:19. doi: 10.11606/s1518-8787.2022056003467 (PMC8973024; doi:10.11606/s1518-8787.2022056003467)
Supplement: Translation

# Translation: Ferramenta de apoio à decisão sobre o rastreamento do câncer de próstata no Brasil

**Authors:** Renata Oliveira Maciel dos Santos, Mirhelen Mendes de Abreu, Arn Migowski, Elyne Montenegro Engstrom

**Affiliations:** I Brasil Instituto Nacional de Câncer José Alencar Gomes da Silva. Rio de Janeiro, RJ, Brasil; II Brasil Universidade Federal do Rio de Janeiro. Programa de Pós-Graduação em Clínica Médica da Faculdade de Medicina. Rio de Janeiro, RJ, Brasil; III Brasil Instituto Nacional de Câncer José Alencar Gomes da Silva. Instituto Nacional de Cardiologia. Rio de Janeiro, RJ, Brasil; IV Brasil Fundação Oswaldo Cruz. Escola Nacional de Saúde Pública Sergio Arouca. Rio de Janeiro, RJ, Brasil

**Keywords:** Neoplasias da Próstata, prevenção & controle, Programas de Rastreamento, Tomada de Decisão Clínica, Uso da Informação Científica na Tomada de Decisões em Saúde, Relações Médico-Paciente

## Abstract

**OBJETIVO:**

Apresentar o processo de desenvolvimento e validação de uma ferramenta de apoio à decisão para o rastreamento do câncer de próstata no Brasil.

**MÉTODOS:**

Estudo com desenho qualitativo-participativo para elaboração de uma ferramenta de apoio à decisão para o rastreamento do câncer de próstata, com a participação de um grupo de homens e médicos inseridos na atenção primária à saúde de 11 estados brasileiros. Realizou-se síntese de evidências, teste de campo e utilização nos cenários clínicos, de modo a adaptar o conteúdo, formato, linguagem e a aplicabilidade às necessidades do público-alvo nos anos de 2018 e 2019. As versões foram avaliadas de forma subsequente pelos participantes, sendo modificada a partir dos dados obtidos.

**RESULTADOS:**

Foi elaborada uma ferramenta inédita no Brasil, com informações sobre os exames utilizados no rastreamento, comparação dos seus possíveis benefícios e malefícios e um infográfico numérico com as consequências dessa prática. Verificou-se utilidade da ferramenta para auxiliar na comunicação entre o médico e o homem no contexto da atenção primária à saúde, além de identificar a necessidade de maior discussão sobre o compartilhamento das decisões nos cenários clínicos.

**CONCLUSÃO:**

A ferramenta foi avaliada como de fácil utilização, objetiva e com pouca interferência no tempo de consulta. É um material técnico-científico, produzido por meio de pesquisa, com a participação do seu principal público-alvo e que se encontra disponível gratuitamente para utilização nos cenários clínicos do Brasil.

## INTRODUÇÃO

O rastreamento do câncer de próstata é amplamente difundido na prática clínica, embora as melhores evidências disponíveis apontem para um desequilíbrio entre os riscos e os possíveis benefícios dessa prática^1^. A realização rotineira do antígeno prostático específico (PSA) e/ou toque retal em homens assintomáticos resulta em muitos diagnósticos e é acompanhada de importantes danos à qualidade de vida do homem^2^.

Esses danos decorrem de resultados falso-positivos, que podem levar à necessidade de uma biópsia para descartar o câncer, podendo ocasionar dor, sangramento e infecção^2^ como consequência desse procedimento. Outros riscos estão relacionados ao sobrediagnóstico e ao sobretratamento, que se caracterizam, respectivamente, a diagnosticar e tratar um câncer que não evoluiria a ponto de ameaçar a vida e tem por complicações mais comuns a disfunção sexual erétil e a incontinência urinária^2,3^, além de efeitos emocionais ao homem e aos familiares.

Considerando essas informações, a recomendação do Instituto Nacional do Câncer (INCA), desde 2008, é que o rastreamento só seja feito em homens que demandam espontaneamente esses exames e após um processo de decisão compartilhada, considerando os riscos e a incerteza sobre os benefícios^4,5^.

A tomada de decisão compartilhada se caracteriza por um processo colaborativo, onde são discutidas as opções de cuidado e suas possíveis consequências, de modo a alcançar a decisão mais adequada ao contexto de vida da pessoa. Essa abordagem é especialmente relevante em situações com algum grau de incerteza na relação entre riscos e benefícios. De modo a facilitar essa abordagem de cuidado, experiências internacionais recomendam a utilização de ferramentas clínicas, denominadas de apoio à decisão, para facilitar a comunicação e deliberação dessas informações. Essas ferramentas são indicadas, principalmente, quando há opções razoáveis em termos de resultados de saúde, as quais as pessoas valorizam de maneira diferente^6^.

As ferramentas de apoio à decisão são encontradas em diversos formatos, incluindo material impresso, vídeos ou dispositivos eletrônicos, que estimulam a participação dos usuários na tomada de decisão, facilitando o aumento de conhecimento sobre a temática discutida^7,8^.

Com intuito de auxiliar na comunicação clínica das implicações do rastreamento do câncer de próstata, prática sensível às preferências individuais, propôs-se construir um material que possa ser utilizado durante a consulta na atenção primária à saúde, nível responsável pela detecção precoce do câncer no Sistema Único de Saúde^9^. A ferramenta é um material técnico de uma instituição federal, sendo disponibilizada e distribuída para as unidades básicas de saúde no Brasil. Dessa forma, o objetivo desse estudo é apresentar o processo de desenvolvimento e validação de uma ferramenta de apoio à decisão para o rastreamento do câncer de próstata no Brasil.

## MÉTODOS

Seguindo as recomendações do *International Patient Decision Aid Standards* e experiências anteriores^10^, uma abordagem de três etapas foi utilizada para desenvolver e validar a ferramenta de apoio à decisão, considerando a avaliação do público-alvo: homens saudáveis e médicos inseridos na atenção primária à saúde. O desenho do estudo foi o qualitativo-participativo, que preconiza a integração dos sujeitos nos processos de produção da pesquisa, contribuindo não apenas com consentimento ou informação, mas modificando sua condução a partir de seus conhecimentos e vivências^13,14^, conforme descrito nas etapas a seguir:

### Etapa 1: Concepção da Ferramenta

A primeira etapa teve o intuito de definir o conteúdo e construir a primeira versão da ferramenta com especialistas. Foi realizada revisão de literatura, com síntese das principais evidências disponíveis sobre o rastreamento do câncer de próstata, considerando ensaios clínicos e revisões sistemáticas sobre a efetividade e as consequências do rastreamento. A busca foi por meio das palavras-chave “câncer de próstata”, “rastreamento” e “apoio à decisão” de forma combinada, na base de dados MEDLINE, em português e inglês, para estudos publicados nos últimos cinco anos, sem filtro de idioma.

Inicialmente, foram selecionadas 1.200 referências que, após leitura do título para verificar o escopo e excluir 247 duplicatas, resultou em 150 artigos. A avaliação desses 150 resumos, selecionou 20 artigos para leitura completa e análise das evidências. Sítios eletrônicos de instituições de pesquisa que desenvolvem ferramentas de apoio à decisão também foram consultados como fontes adicionais e exemplos de infográficos^15,16^. A busca subsidiou a primeira versão da ferramenta, construída a partir da contribuição de seis especialistas das áreas de detecção precoce do câncer e medicina de família.

### Etapa 2: Calibração: Teste Piloto.

O teste piloto buscou adequar o formato e a linguagem ao público-alvo: homens saudáveis em idade madura (aproximadamente 40 anos). A avaliação ocorreu por meio de um grupo focal, com 19 participantes, funcionários de uma empresa pública de energia, localizada na cidade do Rio de Janeiro.

A escolha desse cenário se deu devido à solicitação prévia da empresa à instituição federal para palestras educativas sobre câncer para seus trabalhadores. Na ocasião, foi verificado que a empresa possuía considerável número de funcionários que correspondiam ao critério de inclusão do estudo. Dessa forma, realizou-se convite aberto aos trabalhadores para participação, de forma voluntária, em um encontro de pesquisa, realizado durante o turno de trabalho.

O encontro ocorreu com gravação de áudio, conduzido por dois facilitadores, que trabalharam as questões de pesquisas após leitura individual e coletiva da ferramenta impressa. As contribuições foram analisadas e incorporadas à segunda versão, que seguiu para a próxima etapa do estudo.

### Etapa 3: Viabilidade e Uso da Ferramenta

Nesta etapa, a ferramenta foi aplicada pelos médicos que atuam na atenção primária à saúde a uma amostra de seu público-alvo, com o objetivo de avaliar sua aplicabilidade. Foi solicitado aos médicos que utilizassem a ferramenta em seus atendimentos, quando algum homem demandasse os exames para o rastreamento do câncer de próstata. Como critério de inclusão os médicos deveriam atuar há pelo menos um ano em uma unidade básica de saúde.

Os médicos foram convidados por e-mail, telefone ou aplicativo de mensagens, após divulgação da pesquisa pela rede de contato dos pesquisadores. O convite explicitava os objetivos da pesquisa, os critérios para participação e solicitava aos interessados contatar a pesquisadora principal por e-mail ou telefone. Também foi solicitado que os entrevistados indicassem outros médicos para serem convidados.

Os pesquisadores enviaram 93 convites, resultando em 42 respostas de médicos interessados em receber e testar a ferramenta. Realizou-se contatos semanais com os participantes para acompanhar a pesquisa e solicitar agendamento de entrevista por videoconferência, após no mínimo três utilizações. Nenhum médico informou desistir de participar do estudo, no entanto, 21 não retornaram as mensagens de acompanhamento e seis alegaram não ter tido nenhuma oportunidade para utilizar a ferramenta. Ao final, 15 médicos de 11 estados brasileiros (Espírito Santo, Minas Gerais (2), Rio de Janeiro (2), Paraná, Paraíba (2), São Paulo, Santa Catarina, Rio Grande do Sul, Goiás, Rondônia e Sergipe (2)) foram entrevistados.

Os médicos avaliaram a ferramenta quanto aos seguintes critérios^11,12^:

- Qualidade do conteúdo: examinar a qualidade da informação e a exatidão das evidências científicas;
- Formato: avaliar o layout e o infográfico;
- Aplicabilidade: verificar sua utilização no contexto clínico, uso, aceitabilidade e interferência no tempo de consulta.

As entrevistas foram gravadas, transcritas na íntegra e analisadas a partir dos critérios previamente descritos. As sugestões de mudanças foram incorporadas à ferramenta, que seguiu para a versão final com adequações de texto e formatação, sendo posteriormente publicada como produto técnico de uma instituição nacional especializada em câncer. A versão final foi enviada para dez médicos entrevistados que manifestaram desejo de receber a ferramenta e quatro enviaram comentários.

Esse estudo observou as exigências das Resoluções 466/2012 e 580/2018 do Conselho Nacional de Saúde no que concerne à ética em pesquisa com seres humanos, sendo aprovado pelo CEP/ENSP, CAE: 12165019.4.0000.5240. Todos os participantes forneceram sua concordância por meio da assinatura do Termo de Consentimento Livre e Esclarecido.

## RESULTADOS

O desenvolvimento da ferramenta ocorreu durante os anos de 2018 e 2019 e resultou em um material de duas páginas, composto por textos explicativos sobre o rastreamento do câncer de próstata, os exames utilizados, uma tabela comparativa sobre seus riscos e benefícios, finalizando com um infográfico numérico sobre as consequências dessa prática. O conteúdo técnico foi construído a partir de evidências sobre a efetividade do rastreamento^2,15^, sendo a revisão sistemática mais atual encontrada, 2018^2^, a principal fonte do infográfico.

Seguindo modelos internacionais, o infográfico inicia com a estimativa de rastreamento em 1.000 homens com idade de 55 a 69 anos, acompanhados por 13 anos. Os dados seguem com a taxa de positividade de 10,2% para câncer de próstata, seguido de falso-positivo, que apresentou variação nos estudos entre 10,9% e 19,8%, sendo utilizada a taxa de 17,8%, relatada em cinco centros de pesquisa monitorados pelo *european randomized study of screening for prostate cancer* (ERSPC)^18^. Desse estudo também foram extraídas as taxas de redução de mortalidade específica de 1,3% e a de sobrediagnóstico de 50%^2,16^.

Em relação às consequências do tratamento, optou-se por evidenciar os potenciais danos da prostatectomia radical, por ser o tipo de tratamento mais comum e o indicado como padrão-ouro nas diretrizes brasileiras para diagnóstico e tratamento do adenocarcinoma de próstata^19^. Assim, apresentou-se as taxas de 60% para disfunção sexual, 20% para incontinência urinária e 0,5% para complicações mais graves e morte^2,16^.

Essa primeira versão da ferramenta seguiu para o teste piloto, sendo avaliada em um grupo focal composto por 19 homens, com idade entre 38 e 72 anos (média = 51 anos) e variados níveis de escolaridade e função laboral (Tabela 1).

**Tabela 1:** Perfil dos homens participantes do grupo focal do teste piloto, Rio de Janeiro, 2019.

	n	%
Grupo de homens	19	
Idade		
25 a 29 anos	0	0
30 a 39 anos	4	21
40 a 49 anos	3	16
50 a 59 anos	7	37
≥ 60 anos	5	26
Estado civil		
Casado	12	63
Solteiro	6	32
Viúvo	1	5
Nível de escolaridade		
Fundamental	4	21
Médio	6	32
Superior incompleto	1	5
Superior completo	8	42

A avaliação do grupo de homens gerou modificações na quantidade de informação (diminuição de texto e dados numéricos) e o infográfico foi alterado para uma apresentação mais simples e linear. Alguns termos também foram modificados, de modo a facilitar a compreensão, porém o conteúdo foi considerado adequado e esclarecedor pela maioria dos homens. Perguntas reflexivas ao final do material também foram excluídas por terem sido consideradas de pouca utilidade pelos homens.

*Essas informações são muito importantes, aqui a gente consegue ver bem o que pode acontecer depois de fazer esses exames.* M15

*O material está muito bom, o fluxograma [infográfico] mostra as possibilidades de acontecer alguma coisa… de ter uma complicação, fazer a cirurgia, daí a gente pensa nas possibilidades.* M2

A segunda versão da ferramenta, produto resultante das modificações sugeridas pelo grupo de homens, foi avaliada por médicos que a utilizaram entre três e doze vezes (média = 6), possuíam idade entre 25 e 46 anos (média = 35 anos), mais da metade era do sexo feminino e a maioria com pouco tempo de formação (< 5 anos) (Tabela 2). Devido à heterogeneidade geográfica da inserção dos médicos, que atuavam em áreas urbana (60%) e rural (40%), a ferramenta foi utilizada em homens de diferentes perfis, sendo a maior parte de baixa escolaridade e baixo nível socioeconômico, com idade em torno de 50 anos.

**Tabela 2:** Perfil dos médicos que aplicaram a ferramenta, 2019.

	n	%
Médicos	15	
Idade		
25 a 29 anos	8	53
30 a 39 anos	4	27
40 a 49 anos	2	13
50 a 59 anos	0	0
≥ 60 anos	1	7
Gênero		
Feminino	9	60
Masculino	6	40
Tempo de graduação		
1 e < 2 anos	6	40
≥ 2 e < 5 anos	4	27
≥ 5 e < 8 anos	4	27
≥ 8 anos	1	7
Especialização		
Residência em medicina de família e comunidade	8	53
Outra	1	7
Não possui	6	40
Região		
Norte	1	7
Nordeste	4	27
Sudeste	6	40
Centro-Oeste	1	7
Sul	3	19
Área de atuação		
Urbana	9	60
Rural	6	40

A ferramenta foi considerada útil para a prática clínica e de fácil utilização pela maioria dos médicos (86%), que destacaram a objetividade e o designer como as principais potencialidades. O conteúdo técnico foi qualificado como adequado e correto, não havendo discordância com as informações apresentadas.

A forma de utilização ficou a critério de cada médico, sendo utilizada na tela do computador ou impressa, porém, não foi identificada superioridade entre as duas formas, mesmo quando intercalada pelo profissional. No entanto, alguns relatos sugerem maior praticidade quando a ferramenta está disponível no computador do consultório.

*É mais fácil quando o material já está ali disponível no computador que a gente está atendendo e preenchendo as informações no prontuário… quando o paciente toca no assunto é só abrir e mostrar.* M5

Em contrapartida, dois entrevistados consideraram a ferramenta pouco útil; o primeiro afirma que os riscos do rastreamento não deveriam ser apresentados aos homens e sugeriu excluir essas informações; o segundo afirmou que o médico não precisaria discutir as implicações do rastreamento com o homem, já que essa prática não é recomendada e sugeriu que os dados numéricos poderiam dificultar o entendimento de homens de baixa escolaridade sobre as informações.

*Eu achei bom para o médico, mas achei difícil passar daquela maneira… senti que a informação não é bem absorvida e acabei gastando um pouco mais de tempo para pedir para pessoa olhar… traduzir para ele… pra mim foi bom, me ajudou a sistematizar as informações, mas pro público em si, não achei muito.* M9

Em relação a interferência no tempo de consulta, 53% dos médicos relataram não ter alteração e 33% apontaram diminuição do tempo, afirmando que a ferramenta ajuda a lembrar todos os pontos que devem ser abordados durante a conversa, conforme destacado na fala abaixo:

*É muita coisa que a gente tem que falar para o paciente, tenho que falar de diabetes, tuberculose… quando já está tudo ali, ajuda muito… e como eu tenho pouco tempo de prática, fica mais fácil lembrar tudo que tenho que dizer.* M3

A maioria dos médicos (67%) informou que os homens optaram menos pelo rastreamento após exposição à ferramenta, em comparação aos atendimentos anteriores. Essa mudança foi atribuída à maior compreensão dos riscos, facilitada pelos recursos gráficos e pela credibilidade institucional conferida à ferramenta.

*Faz diferença quando você tem uma instituição por trás. Não sou eu que estou falando, não é só o médico da cidade… Tem uma credibilidade aí, eu fico mais seguro de falar e o paciente vê escrito… faz muita diferença.* M8

Com a ferramenta, os médicos afirmaram ter mais segurança para transmitir as informações sobre os riscos, devido ao respaldo institucional conferido pelo material. Da mesma forma, há relatos de que os homens demonstraram mais confiança em não realizar os exames, quando se depararam com a ferramenta. Essa observação foi acrescida da afirmação de que, sem a ferramenta, os homens repletos de informações favoráveis ao rastreamento, frequentemente se mostravam desconfiados quando apresentados aos riscos, especialmente os que já haviam realizado algum exame anteriormente.

*Tem homem que fica um pouco desconfiado, principalmente os mais velhos, que já fazem esses exames há anos… eu achei que o material me ajudou a mostrar isso para eles, que tem uma ciência nisso.* M6

Por outro lado, a análise das entrevistas também permitiu identificar que mesmo diante da ferramenta a opinião do médico sobre o rastreamento prevalece com grande peso na decisão, uma vez que, suas crenças podem determinar a escolha do homem, conforme destacado a seguir:

*Tem aqui o que acontece se fizer [rastreamento]… os riscos… mas eu já mostro que tem benefício, que eu acho melhor fazer, daí a maioria quer fazer né...* M9

Nesse sentido, como ponto negativo da ferramenta houve menção ao sentimento de frustração do médico quando o homem opta por uma decisão diferente de sua opinião particular e o tempo para incorporar uma nova ferramenta na sua rotina. No entanto, 13 entrevistados (87%) responderam que incorporariam a ferramenta à prática profissional, sendo que dois dos médicos condicionaram seu uso a mudanças na formatação e diminuição na quantidade de texto, que foram posteriormente incorporadas à versão final.

As alterações sugeridas pelos médicos foram a exclusão de um texto explicativo sobre o desenvolvimento do câncer de próstata, uma vez que 93% dos médicos avaliaram como desnecessário, além de alongar o material, e a diminuição de texto no infográfico, colocando maior ênfase nos números e elementos gráficos.

Outra questão identificada pelas entrevistas foi o estranhamento de alguns médicos com uma ferramenta para compartilhar a decisão, sendo necessário inserir na versão final, um texto instrutivo sobre sua finalidade e a importância de discutir os riscos e benefícios com o homem, quando ele demandar o rastreamento do câncer de próstata.[Fig f01002] [Fig f02002]

**Figura 1 f01002:** Versão final da ferramenta de apoio à decisão para o rastreamento do câncer de próstata, página 7.

Fonte: Instituto Nacional de Câncer José Alencar Gomes da Silva. Disponível em: https://www.inca.gov.br/publicacoes/infograficos/ferramenta-de-apoio-decisao-no-rastreamento-do-cancer-de-prostata

**Figura 2 f02002:** Versão final da ferramenta de apoio à decisão para o rastreamento do câncer de próstata, página 8.

Fonte: Instituto Nacional de Câncer José Alencar Gomes da Silva. Disponível em: https://www.inca.gov.br/publicacoes/infograficos/ferramenta-de-apoio-decisao-no-rastreamento-do-cancer-de-prostata

A versão final publicada foi caracterizada pelos médicos como de fácil utilização, linguagem e conteúdo adequados, tendo o layout atrativo e de fácil visualização das informações. Como validação externa, a ferramenta foi avaliada pelos critérios do *International Patient Decision Aid Standards* (IPDAS) para ser incorporada ao *International Inventory of Decision Aids*^20^. A ferramenta atendeu a todos os sete critérios que caracterizam uma ferramenta de apoio à decisão, os quatro critérios para ferramenta de rastreamento, seis dos nove critérios para baixo risco de víeis e 11 dos 13 critérios de qualidade.

## DISCUSSÃO

O processo de elaboração e validação da ferramenta possibilitou demostrar sua utilidade no contexto da atenção primária à saúde no Brasil, a partir da percepção do seu público-alvo. A ferramenta foi considerada de fácil utilização e adequada para a comunicação entre o médico e o homem, no que se refere à deliberação sobre os possíveis riscos e benefícios do rastreamento do câncer de próstata. Dentre as vantagens associadas ao seu uso estão a sistematização das informações, objetividade e pouca interferência no tempo de consulta.

A pouca interferência e até mesmo a diminuição no tempo de consulta, percebida por alguns médicos neste estudo, confere à ferramenta uma importante característica para sua implementação na prática clínica, visto que os profissionais de saúde constantemente enfrentam o desafio de equilibrar seu tempo com muitas tarefas^21^. Revisões sistemáticas que avaliaram ferramentas de apoio à decisão afirmam que, a depender do contexto, do tipo de intervenção e a forma de uso, poderá haver aumento ou diminuição no tempo de consulta^7,8^. No entanto, é necessário ponderar que o tempo gasto em uma comunicação de qualidade, ganha-se em satisfação e melhores resultados em saúde, dentre esses, a diminuição do retorno às consultas pelas mesmas causas^22,23^.

No que se refere à aceitabilidade da ferramenta, apenas dois médicos entrevistados indicaram que não a incorporariam em sua prática clínica. Um refere ser contrário ao rastreamento e, assim, prefere não discutir essa possibilidade com o homem e o outro, por ser favorável, acredita que o homem não deveria ser exposto às informações sobre os riscos. Em ambos os casos, as posições denotam desacordo com a decisão compartilhada para rastrear o câncer de próstata e não a ferramenta em si.

A tomada de decisão compartilhada se caracteriza por um processo interpessoal, no qual os envolvidos colaboram entre si, de modo a alcançar a ação mais apropriada^22,23^. Embora as ferramentas de apoio à decisão facilitem essa abordagem^24^, por si só, não correspondem a todo o processo, uma vez que foi possível perceber algumas barreiras para seu desenvolvimento, tais como a omissão dos riscos do rastreamento ou a resistência do médico em aceitar a opinião do homem, quando diferente da sua.

A despeito de uma intenção protecionista, no que se refere à transmissão de informações complexas, tal postura é contraditória com a ética em saúde, que prima pelo direito individual de conhecer as consequências das intervenções em seu corpo, em especial aquelas com balanço incerto ou desfavoráveis entre os riscos e benefícios^25^.

Ademais, o estranhamento dos médicos com a ferramenta revela a necessidade de estimular a discussão sobre uma maior participação das pessoas nas decisões sobre saúde. Os poucos estudos nacionais sobre a decisão compartilhada apontam para a falta de implementação e institucionalização dessa abordagem nos cenários clínicos, o que demonstra a necessidade de incentivo a essa prática, com ações educacionais voltadas aos profissionais^26^.

Resultados semelhantes são encontrados em estudos de avaliação de ferramentas internacionais de apoio à decisão, tais como a necessidade de investir em recursos visuais, menor quantidade de texto para transmitir o conteúdo^23,24^, dificuldade dos profissionais em partilhar informações consideradas de difícil compreensão e a resistência para a incorporar algo novo à prática clínica^24^.

Como limitações do presente estudo destaca-se a impossibilidade de captar a interferência direta da ferramenta na decisão dos homens, devido à dificuldade em sincronizar a demanda do atendimento com a coleta de dados e a ausência de comparação dos atendimentos com e sem a ferramenta. No mais, recomenda-se cautela na generalização dos resultados por tratar-se de um estudo qualitativo.

Como contribuição considera-se o processo metodológico de desenvolvimento da ferramenta, que envolveu o público-alvo na produção de um material técnico-institucional a ser implementado na atenção primária à saúde no Brasil, que poderá ser replicado para a elaboração de outros materiais, assim como a própria ferramenta em si, que também poderá ser adaptada para outros cenários e países. A versão final da ferramenta se encontra disponível gratuitamente para download no endereço: https://www.inca.gov.br/publicacoes/infograficos/ferramenta-de-apoio-decisao-no-rastreamento-do-cancer-de-prostata, também distribuída impressa em pequena quantidade.

Conclui-se que a ferramenta desenvolvida neste estudo apresenta utilidade para a prática clínica, auxiliando na comunicação entre homens e médicos, além de favorecer a discussão sobre os riscos e benefícios do rastreamento do câncer de próstata. A objetividade, elementos gráficos e a credibilidade foram destacadas como pontos positivos, além da pouca interferência no tempo de consulta.

Por fim, destaca-se que a participação de pacientes e médicos no desenvolvimento da ferramenta possibilitou aproximar o conteúdo e a linguagem das necessidades do público-alvo e, assim, maior correspondência com o material. No entanto, há a necessidade de avaliar a interferência direta da ferramenta na decisão dos homens em estudos futuros, além da efetivação de um plano de implantação para o país, considerando sua heterogeneidade regional no que se refere à organização de serviços de atenção primária à saúde.
